# Rapid Genotyping of Soybean Cultivars Using High Throughput Sequencing

**DOI:** 10.1371/journal.pone.0024811

**Published:** 2011-09-20

**Authors:** Kranthi Varala, Kankshita Swaminathan, Ying Li, Matthew E. Hudson

**Affiliations:** Department of Crop Sciences, University of Illinois, Urbana-Champaign, Illinois, United States of America; French National Centre for Scientific Research-Université Paris-Sud, France

## Abstract

Soybean (*Glycine max*) breeding involves improving commercially grown varieties by introgressing important agronomic traits from poor yielding accessions and/or wild relatives of soybean while minimizing the associated yield drag. Molecular markers associated with these traits are instrumental in increasing the efficiency of producing such crosses and Single Nucleotide Polymorphisms (SNPs) are particularly well suited for this task, owing to high density in the non-genic regions and thus increased likelihood of finding a tightly linked marker to a given trait. A rapid method to develop SNP markers that can differentiate specific loci between any two parents in soybean is thus highly desirable. In this study we investigate such a protocol for developing SNP markers between multiple soybean accessions and the reference Williams 82 genome. To restrict sampling frequency reduced representation libraries (RRLs) of genomic DNA were generated by restriction digestion followed by library construction. We chose to sequence four accessions Dowling (PI 548663), Dwight (PI 597386), Komata (PI200492) and PI 594538A for their agronomic importance as well as Williams 82 as a control.

MseI was chosen to digest genomic DNA based on predictions that it will cut sparingly in the mathematically defined high-copy-number regions of the genome. All RRLs were sequenced on the Illumina genome analyzer. Reads were aligned to the Glyma1 reference assembly and SNP calls made from the alignments. We identified from 4294 to 14550 SNPs between the four accessions and the Williams 82 reference. In addition a small number of SNPs (1142) were found by aligning Williams 82 reads to the reference assembly (Glyma1) suggesting limited genetic variation within the Williams 82 line. The SNP data allowed us to estimate genetic diversity between the four lines and Williams 82. Restriction digestion of soybean genomic DNA with MseI followed by high throughput sequencing provides a rapid and reproducible method for generating SNP markers.

## Introduction

Soybean (*Glycine max*) lines grown in the US were originally introduced from East Asia. A wide range of cultivars are grown in east Asia and have been selected over centuries for yield and suitability to local environment. Earlier domestication of wild soybean involved selection for larger seed and improved nutritional quality. After introduction into the US, commercially grown cultivars were selected for improved yield and biotic/abiotic stress tolerance traits. Studies on diversity of soybean germplasm in the United States have suggested that the introduction and multitude of selection steps may have served as a genetic bottleneck and reduced the genetic diversity within the elite germplasm in the US [1]. An elite US cultivar called Williams 82 [2] was chosen for whole genome sequencing [3].

Breeding practices often involve introgression of desirable traits from a non-elite or wild variety into an elite line. The progeny from such crosses are generally backcrossed to the elite line to recover a near-isogenic line (NIL) with similar yield properties to the elite line and the added trait from the target locus. Molecular markers allow a breeder to rapidly screen a large number of lines for markers associated with the trait, allowing the selection of the molecular marker and thus specific introgression of a single genomic locus. Fine mapping the locus with molecular markers allows the amount of target DNA that will be integrated into the NIL to be reduced. This reduction in linkage drag can also allow reduction in the yield drag often associated with introgression. Therefore the availability of a large number of markers, spread more or less evenly over the genome of a specific exotic line targeted for introgression of a trait, is very valuable. Although fine mapping a locus with tightly linked markers is cost and labor intensive, it might need be done only once per allele of interest. Such an association will, potentially, be applicable in crosses between a different set of parents. The first generation molecular markers were restriction fragment length polymorphism (RFLP) [4], random amplified polymorphic DNA (RAPD) [5], amplified fragment length polymorphism [6] markers or microsatellite DNA markers [7]. Later, Simple Sequence Repeat (SSR) markers provided finer resolution and greater power for cultivar identification [8]–[11]. More recently SNP markers [12]–[14] have grown in stature as an important tool in soybean breeding. Many genetic linkage maps using these marker sets individually or in combination have been constructed to assist breeding [15]–[19]. While SSR markers have a higher distinguishing power between lines, the distribution of SSRs is sparse in the genome and hence may limit the resolution offered in fine mapping. SNP markers, on the other hand have not only been shown to be highly effective in distinguishing lines in the soybean germplasm [14] but are also vastly more common in genomes, especially between two distantly related lines and are thus the predominant markers used in fine mapping. SNP density is expected to be particularly high in the non-coding regions of the genome [20]. One disadvantage of SNP markers is that they are usually available in the form of an array developed for specific genotypes, often not the genotype from which introgression is necessary in a given breeding project. Thus, fewer of the markers on the array are informative in the case of some introgression experiments. We explore the possibility of rapidly and cheaply developing SNP markers for any accession of soybean as referenced against the Williams 82 genome using major recent advances in short read sequencing technologies. These methods potentially allow rapid, low cost genotyping without the high initial costs of developing an array.

In this study we chose four cultivars of soybean (based on the presence of useful resistance traits) to explore a method for exploiting the stated advantage of short read sequencing in generating SNP markers. Dowling is a low-yielding southern US accession of soybean that has proven useful as a source for the Rag1 allele, which provides soybean aphid resistance [20], [21]. Dwight is an elite soybean cultivar often used as the high yielding recurrent parent in breeding [22]. The Komata and PI 594538A accessions carry the Rpp1 and Rpp1-b alleles that confer resistance to soybean rust [23] and are being used in attempts to integrate this trait into commercial lines. Williams 82 was also included to provide a base line for interpreting the experimental results, since it was the source of the DNA used for the reference genome sequence.

Sequencing technologies such as Illumina genome analyzer and ABI SOLiD offer the ideal combination of depth of coverage and frequency of sampling to generate a large set of SNP markers. The approach used in this work is to generate a large number of short reads from genomic DNA of these cultivars and align them to the reference genome. Numerous methods of SNP calling from sequence read data have been developed, each differing in the details of implementation and confidence measures used for calling SNPs. SNPs detected by high throughput sequencing of genomic DNA and called by these programs have the potential to provide very fine resolution of the genomic differences between the cultivar sequenced and the reference assembly, here the Glyma1 assembly [3] of the Williams 82 genome. Owing to reduced selection pressure outside protein-coding regions, a large number of variant SNPs can be expected between the genomes of the cultivars of interest if intergenic sequences are included in the analysis.

Assuming a minimum requirement of three reads covering the base in question to call a SNP and given the size of the *G.max* genome, estimated to be n = 1.1 gigabases (Gb) [24], a random whole genome shotgun sequencing effort will have to be produce at least 3.3 Gb of raw sequence per cultivar to provide sufficient confidence in most SNP calls to produce a high density map. While the cost of producing such large amounts of sequence data has steadily decreased over time, it is nonetheless a substantial investment. Furthermore, sequencing a randomly sheared genomic DNA library in a complicated eukaryotic genome such as *G.max* is estimated to produce a large proportion of reads from the repetitive fraction of the genome. The proportion of reads sampled from a repeated region is directly proportional to the fraction of the genome representing repeats. Up to 60% of the *G.max* genome is estimated to be composed of moderately to highly repetitive elements [3], [25]. The correct alignment of reads sampled from a repeat region is ambiguous by definition due to the presence of many repeating units from a single repeat family. Therefore for the purpose of identifying reliable SNP markers it is desirable to reduce the representation of repeat elements in the sequencing library. It is possible to exclude many repeats by sequencing mRNA in the form of Expressed Sequence Tags and to mine these for SNPs, and this has been done previously in soybean [17]. However, a higher rate of mutation in non-genic sequences, (including both repetitive and non-repetitive elements) is expected compared to the protein-coding regions of the genome, which are more functionally conserved. Thus, the ideal SNP discovery method would exclude repeats while preferentially targeting non-protein-coding DNA. In an earlier survey sequencing effort we characterized the repeat content and composition of the *G.max* genome (Williams 82) [25]. The study also identified the SB92 repeat family as being a predominant repeat that represents close to 3% of the soybean genome. We hypothesized that a method devised to target non-repetitive sequences on the basis of this information would reduce the representation of the repeat content in the sequencing library. During the genome fragmentation stage of library preparation, directed cleavage of DNA by Type II restriction enzymes, as opposed to random shearing, offers an effective way to anchor the start of a short read preferentially to certain sites. Such complexity reduction methods have been successfully applied to alter the genome sampling frequency in multiple organisms [26]. More recent improvements in sequence yield and multiplexing protocols allow a vastly more intricate design to develop high density linkage maps at a population level [27]. We thus deployed a method that targets deep sequencing at Type II restriction enzyme recognition sites, a procedure that has recently been used by others in soybean [28]. In the study presented here, the enzyme choice was determined on the basis of numerical analysis of a prior repeat survey [25] in order to reduce the likelihood of cleavage within repeats and maximize the number of useful sequence reads.

## Results

### Restriction enzyme choice

Choosing an enzyme that does not cleave in any highly repetitive region (as identified previously [25]) for library preparation causes a larger proportion of reads to begin in low-copy regions. The mathematical repeat definition utilized has two critical advantages over traditional repeat masking approaches. First, it identifies previously unknown repeats that have no detectable sequence similarity to known repeats. Second, when applied to a low coverage 454 or other next-generation survey sequencing it can identify repeats that are not normally incorporated into genome assemblies. Highly repetitive regions such as the centromeric, pericentromeric, telomeric and NOR regions are often missing in genome assemblies and hence cannot be masked out. Therefore estimates, of restriction site frequency, derived from an assembled genome sequence will likely be biased by the absence of potentially abundant sites. This repeat identification approach is also useful in informing repeat masking algorithms which are based on the knowledge of known repeats in that organism or related species.

While it is highly unlikely to find an enzyme that does not cleave in any repeated sequence in any given genome, knowledge of repeat composition allows selection of an enzyme that substantially increases the representation of non-repetitive regions in the library. The choice of restriction enzyme to digest genomic DNA was made based on the following criteria: 1. The enzyme should cut often enough in the genome to sample sites at a fairly small physical interval. 2. The recognition site should be present more often in the non-repetitive regions of the genome than the repetitive and 3. The enzyme recognition site should be absent in the extremely abundant 92 bp peri-centromeric repeats CentGm-1 and CentGm-2 [29]. To satisfy the first condition we limited the candidate enzymes to those whose recognition sequence is four to six base pairs. To identify the relative frequency of recognition sites in highly repetitive regions compared to less repetitive regions, it is imperative to identify the highly repetitive fraction of the soybean genome. A whole genome survey sequencing [25], done at 0.7× coverage, of the 1.1 Gb soybean genome was used to identify the highly repetitive component. The Lander-Waterman model [30], originally developed to describe a non-repetitive genome, when applied with these parameters of genome size and coverage predicts that it is unlikely to sample any region repeatedly, and this likelihood decreases exponentially with the depth of coverage. Therefore any overlapping reads seen in such a survey are expected to come from repetitive regions. Based on the model predictions it was estimated that any sequence covered by three reads or more is expected with high confidence to occur in multiple locations in the genome. Therefore all contigs from the non-cognate assembly [25] containing three reads or more were classified as repeats. Any read with no detectable overlap or overlapping with only one other read is assumed to have been derived from unique or low copy number regions of the genome. Restriction enzyme site frequencies were computed independently in the repetitive and low copy number sets of sequences. Each enzyme was then scored on the relative frequency of its recognition site in the non-repetitive set compared to the repetitive one. The enzyme MseI was selected, as it showed the highest bias towards low copy regions while still matching the other two criteria mentioned above. MseI is a type II restriction enzyme with a four base recognition site TTAA and cleaves after the first base, leaving a 5′ TAA overhang. As such, the sites for this enzyme are extremely common in the genome, (around every 100 bp on average). In order to reduce the number of total sites sequenced, nuclear DNA from each accession was digested with MseI and the 100–150 bp fraction from each digestion was sequenced on a single lane of the Illumina genome analyzer. This size-fractionation step causes only those MseI sites located within 100–150 bp of another MseI site to be targeted for sequencing, around 10% of the total number of sites. MseI sites within 100–150 bp of each other occur on average every 1032 bp in the Williams 82 reference genome sequence.

The number of bases covered by at least one read in each of the lines is as follows: Dowling: 55,730,683, Dwight: 57,862,248, Komata: 93,217,361, PI 594538A: 96,473,008 and Williams 82: 57,661,682. The GC composition of reads mapping to Glyma1 ranged from 33 to 39%, which is comparable to the 36.8% GC composition of Glyma1. This implies that the restriction strategy did not introduce a bias towards or against the GC richer or poorer regions of the genome.

### SNP discovery

Sequencing yielded 35 base reads from each of the libraries. The number of reads sequenced from each library varied appreciably, as expected, and hence the amount of sequence coverage is unequal across the libraries (Table 1). Efficiency of restriction site anchoring was tested by counting the percentage of reads that begin with the expected TAA overhang from the restriction digestion (Table 1). Frequency of the trinucleotide TAA in the soybean genome assembly is 53,859,048 i.e., approximately 16.92% of all trinucleotides in the genome. Given this background, if the genome were to be sheared randomly and sequenced, about 17% of the reads are expected to start with the bases TAA. 81–94% of the reads in each of our libraries began with the trinucleotide TAA. Hence a very significant over representation of reads starting with TAA was obtained, thus confirming that the DNA library built from the restriction digested DNA was heavily biased towards true MseI fragments. All reads were aligned to the Glyma1 assembly using the m.a.q. alignment program [31]. The percentage of reads, from each library, which align successfully to the Glyma1 assembly either uniquely and/or in multiple locations are listed in Table 1. The anchoring strategy restricted the sampling sufficiently to increase depth from approximately 0.25× coverage expected from a random shotgun sampling to approximately 4×, thus allowing greater confidence in calling SNPs. The number of SNPs identified from each accession is listed in Table 2. The list of high confidence SNPs described here was generated from a larger set of SNP calls generated by m.a.q. by applying a high stringency filter to increase confidence in the SNP call and reduce false positives. SNPs were filtered to only include calls that were very high confidence: covered by at least 3 reads, minimum consensus quality of 20 for the polymorphic base and two bases on either side, no indels within 6 bp and no more than 2 SNPs in a 10 bp window. This set of parameters ensures that no SNP calls are based exclusively on repetitively mapped or poorly aligned reads. The number of SNPs discovered correlates with the expected genetic distances of these accessions, since Dwight has a known higher coefficient of parentage with Williams 82, and Williams 82 is the line from which the reference sequence was generated. Dwight and Williams 82 share a common parent [2], [22].

**Table 1:** Efficiency of sampling strategy.

Variety	Dowling	Dwight	Komata	PI 594538A	Williams 82
Total Reads	7625036	11764203	8452272	9309921	7583486
% of reads starting with TAA	87	94	81	86	94
% of reads mapped	95	96	88	91	97
Tagged 35mers	2148006	2410953	3310171	3506523	2053175
low copy %	57	43	69	72	55

Reads were mapped to the Glyma1 reference assembly at at least 90% identity. Tagged 35mers represents the number of unique 35 bp frames in the genome that are covered by at least 1 read. Low copy reads are those that map to less than 5 loci in the genome.

**Table 2:** High confidence SNPs and SNP density.

Variety	Dowling	Dwight	Komata	PI 594538A	Williams 82
Mean Coverage	4.56	6.85	2.81	3.07	4.45
Filtered SNPs	6019	4294	12727	14550	1122
MDBS	626	904	609	649	4168
MDAS	17.1	17.33	6.59	8.26	12.89

The SNP density between each line and the reference assembly is measured as the total number of good quality bases resequenced in that line divided by the number of high confidence SNPs. This measure is called Mean Distance Between SNPs (MDBS). Mean Depth At SNP (MDAS) assesses confidence measured as number of reads aligned at the SNP position.

**Figure 1:**
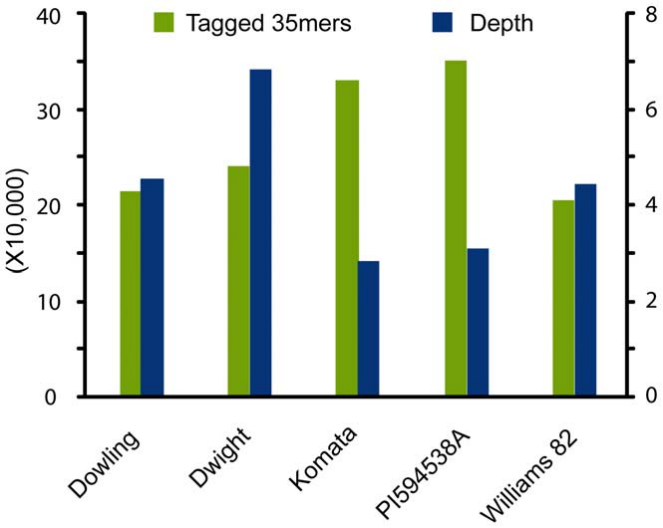
Sequence coverage at tagged sites across varieties subjected to genotyping by sequencing. Average depth of coverage across all bases covered by resequencing reads is shown by blue columns with the value indicated by the vertical axis on the right. The variation in depth observed between libraries is a combination of variation in the amount of reads obtained in a sequencing run and the number of loci tagged by a read in that library. The total number of 35mers from the genome that were tagged by at least one read is shown by the green columns. The vertical axis on the left depicts the number of sites tagged (in tens of thousands).

**Figure 2:**
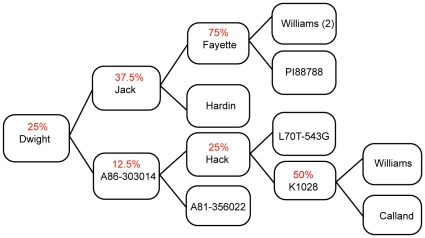
Pedigree of Dwight. The Dwight variety of soybean was produced by crossing Jack and an experimental line. Following the parentage back 3 generations reveals that the Williams line served as an ancestor on both sides of the cross and was used as recurrent parent to varying degrees. Numbers in parenthesis indicate the number of times a line was used as a recurrent parent. Based on the parentage, the proportion of the genome that is expected to be from Williams is indicated in Red. Lineage is depicted with the progenitors to the right.

To express the degree of diversity between lines we used Mean Distance Between SNPs (MDBS), the average distance between polymorphisms within regions sequenced in this experiment. This was calculated by computing the number of bases covered by at least three reads and dividing that by the number of SNPs, called at high confidence relative to the reference genome, without accounting for the base quality. Since the average quality for bases is comparable between the 5 libraries (data not shown) we estimate that any local biases in mapping quality will even out over the genome. After normalization the MDBS is almost even between the Dowling, Komata and PI 594538A lines but shows a marked increase in Dwight and is very large in Williams 82, as expected (Table 2). Even though the raw count of SNPs is higher in the two East-Asian accessions (Komata and PI 594538A as compared to Dowling) the number of sites sampled in them is significantly higher (Figure 1) thus providing an important correction for the SNP density estimation. Dwight is expected to share at least a quarter of its genome with Williams (Figure 2). This common parentage explains the lower diversity between Williams 82 and Dwight.

**Figure 3:**
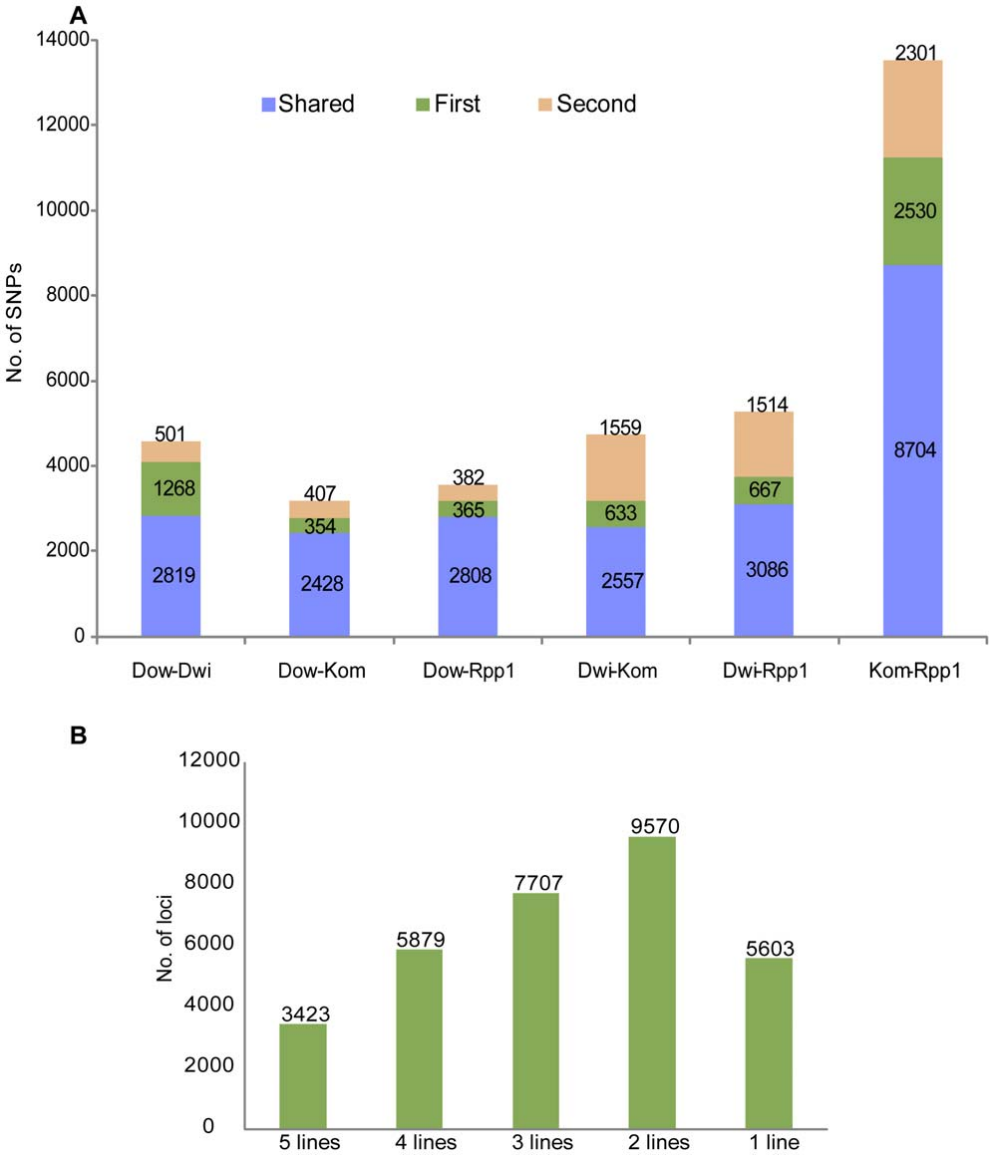
SNP loci genotyped across accessions. A. All high-confidence SNP loci identified from two accessions were pooled and filtered to retain only those loci which were genotyped in both. Loci were then classified based on the the presence of the SNP into 3 categories: 1. SNP was observed in both accessions when compared to the reference sequence, 2. SNP was observed in the first accession but not the second and 3. SNP was observed in the second accession but not the first. The sum of SNP loci in categories 2 and 3 is the number of loci detected as polymorphic between these two accessions. B. The number of loci genotyped with 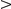 = 3 reads in 1–5 lines are shown.

**Figure 4:**
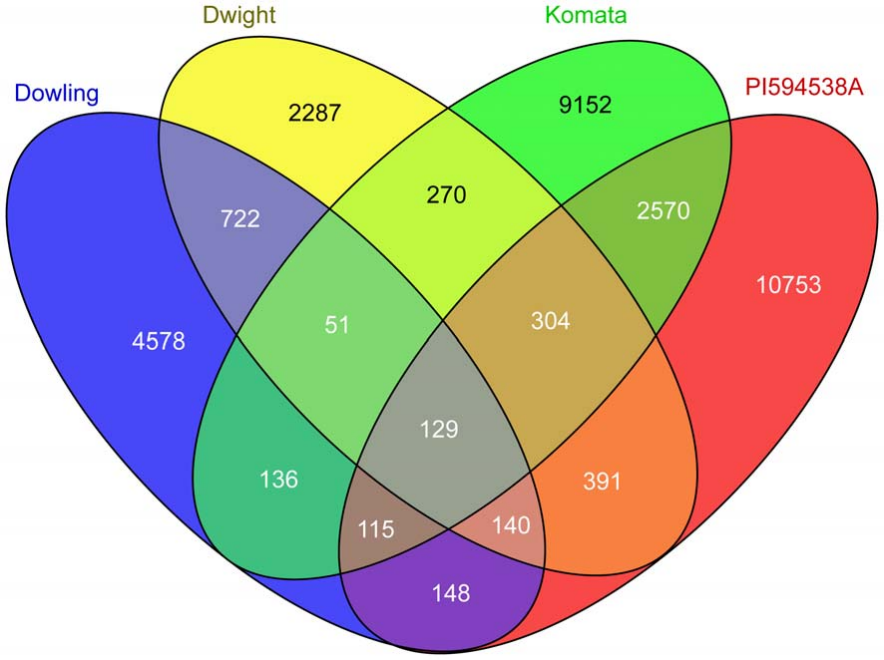
SNP loci shared between accessions. The number of high-confidence SNP loci shared between accessions is shown. A large proportion of SNP loci from any given accession seem unique to that accession. This unique portion is most likely an overestimate since the corresponding loci were simply not genotyped in the other accessions.

The SNPs most informative from a molecular breeding perspective are those that are polymorphic between the lines of interest. To assess the number of informative SNPs between the various accessions sequenced here, the predicted genotype of SNP loci in all pairwise comparisons were studied. SNP loci that were successfully genotyped in both accessions being compared were classified into 3 categories: 1. SNP shared in these two accessions when compared to the reference, 2. SNP is polymorphic between the First accession and reference but not the second and 3. SNP is polymorphic between the Second accession and reference but not the first (Figure 3A). The union of sets 2 and 3 from the above classification is the set of SNP loci that are polymorphic between the two accessions under comparison. Figure 3B shows the number of SNP loci that were genotyped across 1–5 accessions. The linear increase in number of SNP loci genotyped relative to the decrease in number of accessions required is fairly reflective of the incomplete sampling of MseI sites obtained in this study. Nonetheless the number of SNP loci genotyped only in one line is low implying that there is a fair degree of overlap among the sites genotyped across accessions. The overlap between identified SNP loci can also be visualized as a 4-way venn diagram as in Figure 4. Komata and PI 594538A were sampled more extensively and hence show a higher number of SNPs. The various overlaps show the number of SNPs shared between those sets of accessions. The largest such overlap is again between Komata and PI 594538A, partly due to sampling and partly due to their expected genetic distance from the north american accessions. Additionally these two east asian accessions might have some degree of shared parentage.

To test the predicted high-confidence SNPs, we independently sequenced the SNP loci using traditional Sanger sequencing. Twenty SNP loci, with good quality flanking sequence, that are polymorphic between Komata and the reference genome were chosen for confirmation. Sequence around this region was extracted from the Glyma1 assembly, and used for primer design. Of these twenty regions, two failed to amplify with the designed primers. The eighteen other primer sets amplified a single region, as evidenced by a single band on a gel. Using Sanger sequencing, we confirmed the predicted SNP in these eighteen loci. In two cases additional SNPs in the vicinity that had not passed the high confidence SNP filter were also confirmed, implying that the SNP density estimate we arrived at is likely to be a conservative estimate of the true variation between these lines. A further 15 SNP loci that are polymorphic between Dowling and the reference were chosen randomly. Nine primer sets, designed from the reference genome, amplified a fragment from Dowling genomic DNA which was then submitted for sequencing by sanger method. Eight of the nine predicted SNPs were validated in this set implying that our overall false SNP discovery rate is less than 5%.

### Heterogeneity in Williams 82

**Figure 5:**
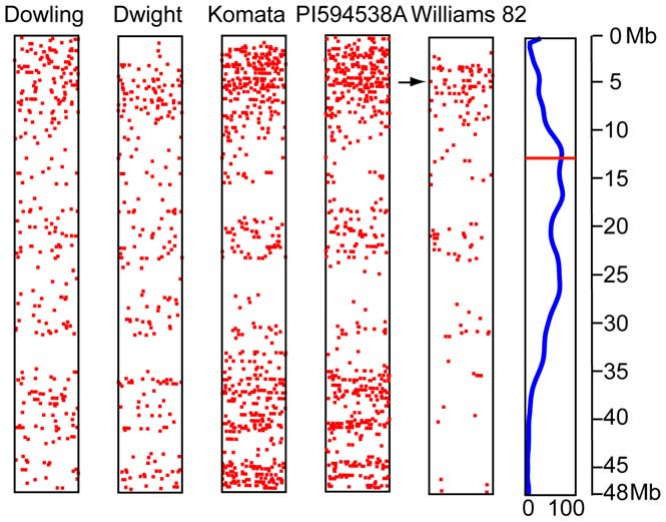
SNPs polymorphic between each variety sequenced and the Glyma1 assembly of chromosome 3. High confidence SNPs called from 3 or more reads aligned against the Glyma1 reference assembly of Gm03 (Linkage group N) are shown. At the right edge of the image, the centromere is indicated by a red bar and % repetitive content of 100 Kb blocks (ranging from 0–100%) is plotted as a bezier curve. Note that repetitive sequences (predominant in the centromere) prevent unique mapping of sequence reads and thus show substantially reduced SNP density. Scale is in megabases (Mb) of physical distance. SNPs occurring in a one million basepair (MB) bin are dithered, left-to-right, along the X axis based on the position of SNP within that bin. The presence of a large number of SNPs and their non-random distribution on Gm03 for the Williams 82 data suggests that the Williams 82 line carries significant portions of the non-recurrent parent Kingwa. The distribution of SNPs in other lines shows a density proportional to sampling frequency and shared parentage, while the Williams 82 line shows higher diversity around the 5MB mark of Gm03 (arrow).

**Figure 6:**
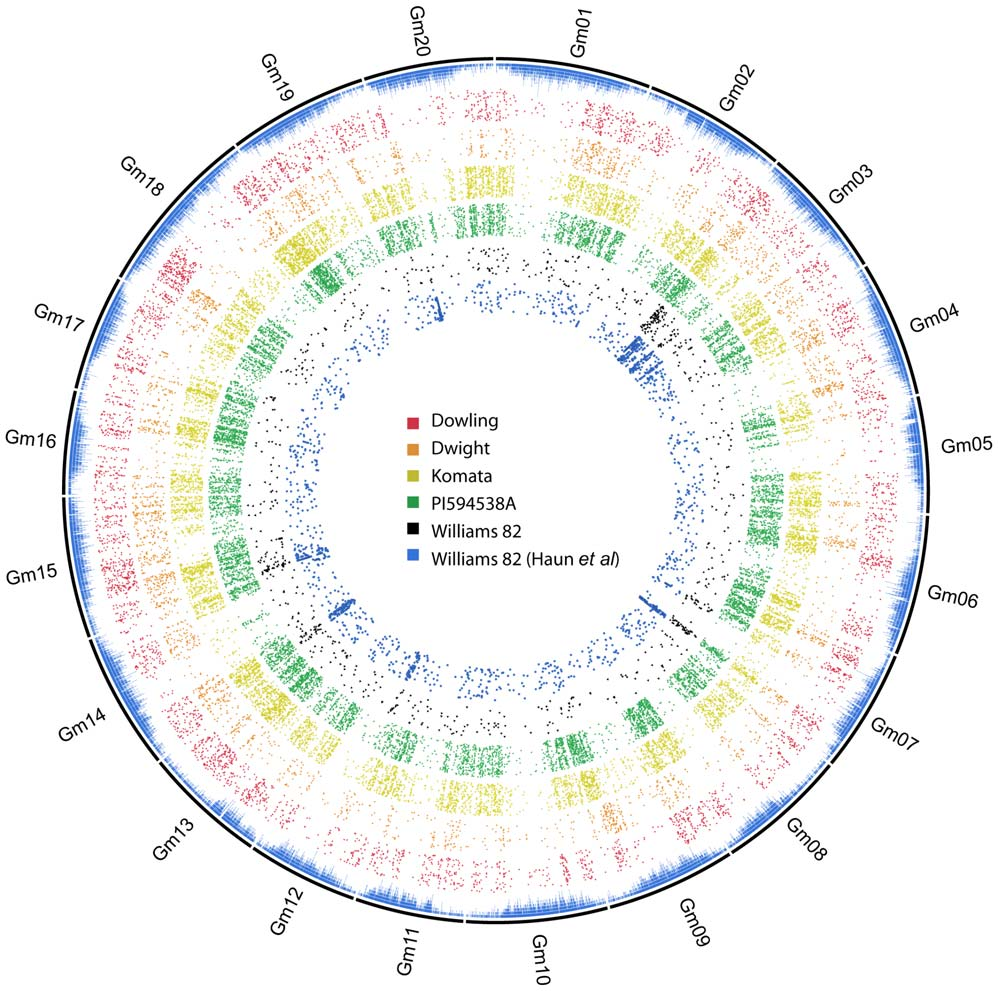
SNPs between resequenced accessions and the Glyma1 assembly. High confidence SNPs called by aligning reads against the reference assembly are shown in concentric rings. The outer most ring depicts the known repeat elements as stacked blocks. The subsequent rings each depict the position of high-confidence SNPs from the lines Dowling, Dwight, Komata, PI 594538A and Williams 82 consecutively. The inner-most ring depicts SNPs identified from exome capture sequencing of two Williams 82 individuals by Haun et al. (Data kindly provided by Robert Stupar). Genomic regions rich in repeats, as shown by higher stacks of blocks on the outer-most ring, render themselves poorly to unambigous read alignment and hence SNP calling. Outside these regions SNPs are distributed evenly across the genome in all the accessions sequenced except for Williams 82. Data from our study and Haun et al concur on the regions of high heterogeneity restricted mostly to Gm03 but also on Gm07, Gm14, Gm15. Haun et al. data additionally identifies a heterogenous region at the start of Gm20 that was not observed in our data.

Williams 82 was created by crossing Williams and Kingwa accessions of soybean [2]. The expected proportion of Kingwa genomic DNA in the Williams 82 genome is 1.75% based on the pedigree information, with most of it expected to be centered around the Rps1 locus on Gm03 [32], [33]. We sequenced Williams 82 to serve as a negative control for the experiment in anticipation of having to adjust SNP calling parameters for the inherent biases/errors in Illumina short read sequencing. Ideally no SNPs should exist between the Williams 82 sequencing run and the reference genome at the correct level of stringency required to remove false positives, since our genomic DNA was derived directly from the reference allele acquired from the USDA Soybean Germplasm collection. Despite repeatedly increasing the stringency level we continued to observe SNPs between the resequenced Williams 82 library and the reference Glyma1 assembly (Figure 5 and 6). Additionally we observed a 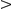
95% confirmation rate by Sanger sequencing for the SNPs identified between the Komata reads and the reference assembly at the default stringency level. Thus the SNPs detected between our Williams 82 reads and the Glyma1 reference assembly are highly unlikely to be the result of errors either in our sequence or in the Glyma1 sequence.

In parallel with our study, Haun et al. [34] found that the Williams 82 cultivar contains a significant level of residual genetic variation. We found that many SNPs are detected between the Williams 82 used for this study (acquired from the Soybean Germplasm Collection at the University of Illinois) and the reference genome sequence [3]. This is likely a result of genetic variation between the plants used for sequencing of the reference and the Germplasm Collection line used in this study.

### SNP distribution

The SNP positions for each accession were plotted on the twenty soybean chromosomes to visualize their distribution (Figure 6). Large gaps in the distribution coincide with the highly repetitive centromeric and pericentromeric regions of the chromosomes [3]. Among the four sequenced accessions (excluding the Williams 82 control) SNP distribution is fairly even in the low copy regions, implying a lack of bias in sampling (Figure 5) [35]. The observed even distribution of SNPs discovered using this method in conjunction with the consistent mean SNP density of 600 base pairs, within sequenced regions, among the accessions makes this method of SNP discovery an excellent tool for marker development in soybean. The SNP density obtained in this study for mapping purposes, measured as the median distance on the chromosome between any two SNPs discovered using this method, is 46.6 Kb for Dowling, 44.4 Kb for Dwight, 19 Kb for Komata and 16 Kb for PI 594538A. All of these distances are short enough to be used for extremely fine genetic mapping. In contrast to the data for the other accessions, the SNP distribution plot for Williams 82 SNPs relative to the reference Williams 82 assembly clearly shows regions of high and low diversity (Figure 2) between the genotype that was sequenced and the reference. Interestingly the Rps1 gene cluster maps to Gm03 at approximately position 5,000,000 in the Glyma1 assembly [33], a region showing the highest SNP density among all chromosomes between the reference sequence and the genotype sequenced in this study (Table S1). The same region was identified as a location with high variation between individuals of Williams 82 in another study [34] (Sequence data kindly shared by Robert M Stupar in a personal communication). In addition high SNP density regions are seen in Gm07, Gm14 and Gm15. These regions likely correspond with the portions of the Kingwa genome retained in Williams 82 during back-crossing and subsequent selection.

### Transposable element families

**Figure 7:**
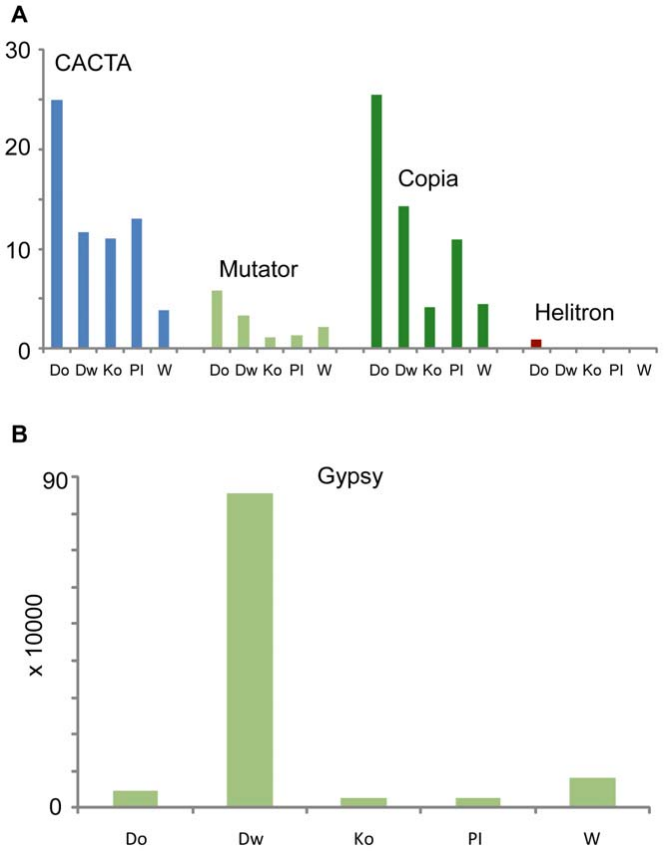
Transposon family divergence. All reads were aligned to the soybean transposable element database (soyTEdb) and grouped based on the transposon family they match. The number of reads assigned to each family was normalized to the total number of reads from that library to allow comparison across lines. Abbreviations for soybean genotypes: Do = Dowling, Dw = Dwight, Ko = Komata, PI = PI 594538A, W = Williams 82. A) CACTA and Copia families show a significant expansion in the Dowling accession. B) Elements of the Gypsy family have substantially increased numbers in the Dwight genome and are increased to a lesser extent in the Williams 82 genome.

A number of reads from each library mapped to known transposable elements (TEs) in soybean. Reads were assigned to TE families by mapping them to the elements listed at soyTEdb [36]. The number of reads matching TE families was normalized to the total number of reads mapping to the reference genome generated from each accession. Since some reads matched to many members of a TE family, the contribution of a read matching multiple TEs was divided by the number of elements the read mapped to. Weighted read counts were summed up for each family of TEs based on the family-level annotation from soyTEdb. Interestingly, the Gypsy family of elements shows higher numbers in the Williams 82 and Dwight genomes relative to the other accessions sequenced (Figure 7). The other noticeable expansion is in the Copia and CACTA families in the Dowling genome. These results indicate evidence for variability in TE content between soybean accessions.

## Discussion

Deep sequencing of reduced representation libraries from genomic DNA provides a rapid and relatively inexpensive method of generating markers in lines of agronomic interest. Restriction digestion of genomic DNA offers an excellent way of creating reduced representation libraries. Typically, the restriction enzyme used for digestion is chosen using a general strategy. One such strategy is to use a methylation sensitive enzyme [37]. This approach preferentially targets single-copy sequences, but also targets conserved protein-coding sequences where SNPs are rarer (a disadvantage for less diverse crops such as soybean). It also requires complex procedures to reduce the size of the restriction fragments to a suitable size for Illumina sequencing [38]. An alternative approach is to empirically pick one, or cocktail of few enzymes that give the desired result based on experimental digestion of genomic DNA. Both strategies have been employed with success in plant genomes [39], [40]. In species where the approximate genomic repeat composition is known, it is possible to apply a more rational strategy of choosing a restriction enzyme that provides both an increased depth of sampling and an intentional bias towards the non-repetitive regions of the genome.

In the case of *Glycine max* the enzyme MseI works exceptionally well at reducing the genome complexity sufficiently to allow SNP discovery, while also preferentially targeting intergenic DNA as a result of its lower GC content. A recent study used CviRI digested DNA, based on an in silico analysis of the draft genome and annotated repeat elements, to identify SNPs between Forrest and Williams 82 [28]. Our method of identifying site frequency in the low and high copy regions indicates that CviRI has a frequency ratio of 0.88∶1 in low∶high copy genomic DNA, which compares unfavorably to the 1.23∶1 ratio for MseI. We believe this was the result of our using the mathematically defined repeats by non-cognate assembly of a genome survey [25] rather than using annotated repeats from a genome sequencing project. Mathematically defining repeats is likely to identify more repeats than sequence annotation due to its power to overcome the need to detect sequence similarity across species, and the fact that tandem repeats are often excluded from genome assemblies. A similar strategy can in principle be followed for genotyping multiple accessions of any other crop species with a reference genome and known repeat composition. Even in unsequenced genomes where a survey sequence is available to detect repeat sequences, this method can still be applied, since the length of the restriction site roughly determines the mean distance between such sites in the genome.

With the falling costs of short read sequencing and coupled increases in the number of sequencing reads produced per run, such a deep sequencing strategy is likely to be the most rapid and, perhaps, even the more economical method to generate a large amount of SNP markers for any new accession of interest to plant breeders. A single lane of Illumina sequencing, at the time of this manuscript being written, will likely tag the vast majority of MseI sites in the genome with sufficient depth to allow high confidence SNP detection and provide a very high density SNP map for several accessions using barcoded libraries, yet would still likely be insufficient for full whole-genome resequencing of a single line. In such an experiment the cost per accession is expected to fall further. At the estimated SNP density of 600 bp such genotyping should allow fine mapping a trait of interest down to a very small interval. In specific regions of interest, where a higher density of SNPs is needed, the SNP filter stringency can be lowered accordingly at the cost of increasing the false positive rate (which, as we indicate here, is very low for the procedure as described). Selection based on such markers will facilitate high throughput genotyping of progeny to select for traits of interest. High resolution mapping will also allow reduction of the yield drag often introduced in such crosses by allowing genotyping to select the progeny with least amount of DNA from the lower yielding parent.

There was a noticeable increase in the number of sites sampled in two libraries out of the five. This difference can most likely be ascribed to small differences in the efficiency of digestion between the different DNA samples or in the fraction of genome obtained during the size selection from gel. While this variation suggests that great care must be taken during those steps, the result still provided sufficient sampling from all libraries to enable SNP calling.

Our survey also detected significant residual variation between different sources of the cultivar Williams 82 (the source used for the reference genome sequencing and the soybean germplasm collection). This is a violation of a perhaps unrealistic assumption made historically by many crop biologists, that varieties of an inbred selfing crop such as soybean should be almost completely homogeneous and homozygous. Our results on variation within Williams 82 confirm those of Haun et al. [34] who found that both SNP and copy number variants exist within this line using different techniques to those used in this study. Our Williams 82 DNA was prepared from pools of several individuals, whereas that of Haun et al. was prepared from individual plants. Therefore while we are not able to determine the extent of variation between the individual plants derived from seed from the germplasm collection, we have determined in the case of many of our polymorphisms that the reference genome contains an allele not found in any of the individuals in our pool.

We conclude that individual plant lines must be closely examined using this or a similar genotyping technique to ensure that within-cultivar variation does not cause errors in experiments involving genetic comparison (particularly those that involve the creation of variation using chemical mutagens, such as TILLinG). For important crop species with large germplasm collections or novel plants being introduced into agriculture this method provides a rapid, economical and easy protocol to catalog diversity and generate molecular markers. The advent of longer sequence reads makes this technology also potentially applicable to organisms with unsequenced genomes.

## Materials and Methods

### Restriction enzyme selection

Survey sequencing data [25] was used to classify soybean sequences into a low copy set and a highly repetitive set. The Lander-Waterman model [30] for a non-repetitive genome predicts the number of contigs expected to be formed by an overlap of n reads for a given size of the genome and coverage obtained. Fitting this model to the size of soybean genome and coverage obtained in that study predicted that there should be almost no contigs formed by overlap of 5 reads or more by random chance. The non-cognate assembly showed contigs far exceeding the number of expected contigs beginning at n = 3. Therefore all contigs formed by an overlap of 3 reads or more were defined as sequences with a high copy number for the purposes of this study. This set was composed of 20,670 contigs and all reads in these contigs were extracted to form the repeat sequence set (n = 384,339). Conversely all single reads and reads with a single overlapping read were classified as low copy sequences. Approximately 333,000 reads fell in to the low copy set. The, fortuitously, comparable number of reads in each set removed the need for any normalization of site frequencies. Type II restriction enzymes with a recognition site length of four or six were selected from REBASE [41] and grouped by site. To ease data analysis at later stages all recognition sites with ambiguous bases were removed. In addition all enzymes that do not have a defined sequence at the cut site were removed. To avoid sampling the extremely abundant 92 bp repeat, all enzymes that would cut this repeat were also disqualified. Frequency of each site was then computed in the two sets of sequences. The remaining enzymes were ranked based on the ratio of site frequency in the low copy read set versus repeats. MseI, a type II restriction enzyme with recognition site TTAA, emerged as the best enzyme on this list. The raw site count in the low copy set was 489,740 versus 397,173 in the repetitive set giving a frequency ratio of 1.23 in favor of low copy sequences. MseI cuts the recognition site TTAA leaving a 5′ overhang of TAA.

### Plant material

Seed for each soybean line described in the text (Dowling (PI 548663), Dwight (PI 597386), Komata (PI200492), PI 594538A and Williams 82 (PI 518671)) was obtained from the USDA soybean germplasm collection. Plants were grown in pots in a temperature- and light-controlled greenhouse in long day (18 hr) light conditions for 4–12 weeks. Young leaf and stem tissue, tips of branches with at least two visible leaves, was collected from four to six individuals for each line.

### DNA extraction and digestion

10–20 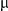
g of Nuclear DNA, extracted from all five lines according to protocols described in Swaminathan et al. [25] was subjected to complete digestion with MseI. The digest was end-repaired with T4 DNA polymerase run on a 3% low melting point agarose gel. The size fraction from 100–150 bp was electroeluted using Spectrapore dialysis tubing (MW cutoff 3500) and precipitated. 200–500 ng was sequenced by Illumina (Hayward, CA).

### DNA sequencing

After library construction for Illumina sequencing, each library was loaded onto one lane of the sequencing flow cell. Sequencing was done on the Illumina GAI genome analyzer system, performed for 35 cycles and bases called. Each library was sequenced twice, except Dwight (sequenced three times), to satisfy quality criteria. Sequence and quality data was obtained in fastq format. All raw data from the sequencing runs was deposited into the SRA at NCBI. The individual runs can be accessed from NCBI using the accessions SRR111923–SRR111933.

### Mapping to Glyma1

Reads were aligned to the Glyma1 version of the soybean genome assembly. Maq (v 0.7.1) was used to align the reads to the genome using the maq.pl script with easyrun option.

### SNP calling

SNP calling was done as part of the easyrun option for maq. Additional stringency levels were tested by running maq.pl with the SNPfilter option and varying the cut off parameters for minimum mapping quality at SNP position and in a 6 bp window around it. These changes did not change the number of SNP calls appreciably. Increasing the minimum depth required to call a SNP decreased the number of SNP calls substantially, especially in the Komata and PI 594538A lines. The SNPs reported in this report were obtained at the default maq parameters of d = 3, -n = 20, -q = 20, -w = 5, and N = 2. All high confidence SNP loci and allelic information were deposited in NCBI's dbSNP database (See Table S2).

### SNP verification

Primers for SNP verification were designed using an in-house script. Komata genomic DNA extraction was performed as described earlier. PCR was performed with Ex Taq (Takara, Japan) according to the product menu. The reactions were first heated at 94C for 5 min, followed by 35 cycles using a 30 seconds denaturation step at 94C, a 30 seconds annealing step at 58C, and a 1-min extension step at 72C. An additional 7-min extension step at 72C was added after 35 cycles. PCR products were analyzed on a 1% agarose gel. In all cases that the PCR product showed as a single band in the gel, the Qiagen PCR purification kit (Qiagen, CA, US) was used to purify the PCR product for sequencing. Sanger sequencing reaction of the PCR product was performed with BigDye Terminator v3.1 Cycle Sequencing Kit (Applied biosystems, CA, USA) according to the manual with the forward primer used in PCR reaction. BigDye reactions were submitted to Keck Center, UIUC for purification and capillary electrophoresis. Sequences were analyzed and compared to Glyma1 genome sequence using Sequencher (Gene codes, MI, US).

## Supporting Information

Table S1 **List of SNPs from each line sequenced with their genomic position according the soybean reference assembly (Glyma 1.09).**
(XLS)

Table S2 **dbSNP accession numbers for all SNP loci.**
(TXT)
